# Chondroprotective Effects and Mechanisms of Dextromethorphan: Repurposing Antitussive Medication for Osteoarthritis Treatment

**DOI:** 10.3390/ijms19030825

**Published:** 2018-03-12

**Authors:** Liv Weichien Chen, Feng-Cheng Liu, Li-Feng Hung, Chuan-Yueh Huang, Shiu-Bii Lien, Leou-Chyr Lin, Jenn-Haung Lai, Ling-Jun Ho

**Affiliations:** 1 Graduate Institute of Life Science, National Defense Medical Center, Taipei 11490, Taiwan; mslivcat@gmail.com; 2 Institute of Cellular and System Medicine, National Health Research Institute, Zhunan, Miaoli 35053, Taiwan; lifeng@nhri.org.tw (L.-F.H.); 000126@nhri.org.tw (C.-Y.H.); 3 Rheumatology/Immunology and Allergy, Department of Medicine, Tri-Service General Hospital, National Defense Medical Center, Taipei 11490, Taiwan; lfc10399@yahoo.com.tw; 4 Department of Orthopaedics, Tri-Service General Hospital, National Defense Medical Center, Taipei 11490, Taiwan; LSB3612@yahoo.com.tw (S.-B.L.); lchlin66@hotmail.com (L.-C.L.); 5 Department of Rheumatology, Allergy and Immunology, Chang Gung Memorial Hospital, Lin-Kou, Tao-Yuan 33305, Taiwan; 6 Graduate Institute of Clinical Research, National Defense Medical Center, Taipei 11490, Taiwan

**Keywords:** dextromethorphan, osteoarthritis, chondrocyte, MMP-13, collagen-induced arthritis

## Abstract

Osteoarthritis (OA) is the most common joint disorder and primarily affects older people. The ideal anti-OA drug should have a modest anti-inflammatory effect and only limited or no toxicity for long-term use. Because the antitussive medication dextromethorphan (DXM) is protective in atherosclerosis and neurological diseases, two common disorders in aged people, we examined whether DXM can be protective in pro-inflammatory cytokine-stimulated chondrocytes and in a collagen-induced arthritis (CIA) animal model in this study. Chondrocytes were prepared from cartilage specimens taken from pigs or OA patients. Western blotting, quantitative PCR, and immunohistochemistry were adopted to measure the expression of collagen II (Col II) and matrix metalloproteinases (MMP). DXM significantly restored tumor necrosis factor-alpha (TNF-α)-mediated reduction of collagen II and decreased TNF-α-induced MMP-13 production. To inhibit the synthesis of MMP-13, DXM blocked TNF-α downstream signaling, including I kappa B kinase (IKK)α/β-IκBα-nuclear factor-kappaB (NF-κB) and c-Jun N-terminal kinase (JNK)-activator protein-1 (AP-1) activation. Besides this, DXM protected the CIA mice from severe inflammation and cartilage destruction. DXM seemed to protect cartilage from inflammation-mediated matrix degradation, which is an irreversible status in the disease progression of osteoarthritis. The results suggested that testing DXM as an osteoarthritis therapeutic should be a focus in further research.

## 1. Introduction

The rising prevalence of osteoarthritis (OA) is a major medical concern worldwide [1]. Although the factors causing arthritis vary, cartilage damage exists in both OA and rheumatoid arthritis (RA) [2]. Cartilage is a major component of joints and has a buffering role to relieve the stress applied to the joints. Chondrocytes, which are the only cell type found in the cartilage, produce collagen and glycosaminoglycan that form a concentrated and highly coordinated extracellular matrix (ECM), which plays critical roles in OA development [3]. Among ECM, manipulating collage II provides a potential implication for cartilage therapeutics [4].

The inflammatory process results in overproduction of metalloproteases that leads to damage of the ECM and joint instability. The pro-inflammatory cytokines, such as tumor necrosis factor-α (TNF-α) and interleukin (IL)-1, play critical roles in inflammation-mediated joint damage by promoting the production of metalloproteases, chemokines, and other inflammatory mediators [5]. Elevated concentrations of TNF-α in synovial fluid have also been demonstrated in patients with knee OA disease progression [6].

Dextromethorphan (DXM), which is considered an antagonist of *N*-methyl-d-aspartate (NMDA) receptors, has been commonly recognized and used as an antitussive agent with very limited safety concerns and no addictive effects, even with high dosage and long-term usage [7]. Its potential applications for neurological and psychiatric disorders such as depression, stroke, traumatic brain injury, and seizure have been extensively discussed [7]. In support of the usefulness of DXM in neurological disorders, DXM treatment decreases the number of immune cells entering the spinal cord and provides therapeutic benefits in experimental autoimmune encephalomyelitis [8]. Aside from neurological disorders, DXM has been shown to ameliorate atherosclerosis-associated chronic inflammation disease progression by reducing oxidative stress [9]. Strikingly, DXM can enhance glucose tolerance in mice, suggesting its potential in treatment against diabetes [10].

Choosing appropriate disease-modifying drugs for OA therapeutics remains a great challenge [11]. The safety profiles of DXM and its broad immunomodulatory effects stimulate our interest for repurposing its other clinical applications. In the present study, we investigated its potential as a disease-modifying anti-OA drug. Here, we demonstrated that DXM preserved chondro-protective effects by modulating inflammation as well as altering the synthesis of matrix-degrading enzymes to prevent the destruction of cartilage. From both in vitro cell system and in vivo mice model studies, we suggest that repurposing DXM to be one of the choices for treatment of OA may warrant clinical trials.

## 2. Results

### 2.1. DXM Counteracted TNF-α-Mediated Reduction of Col II

**Figure 1:**
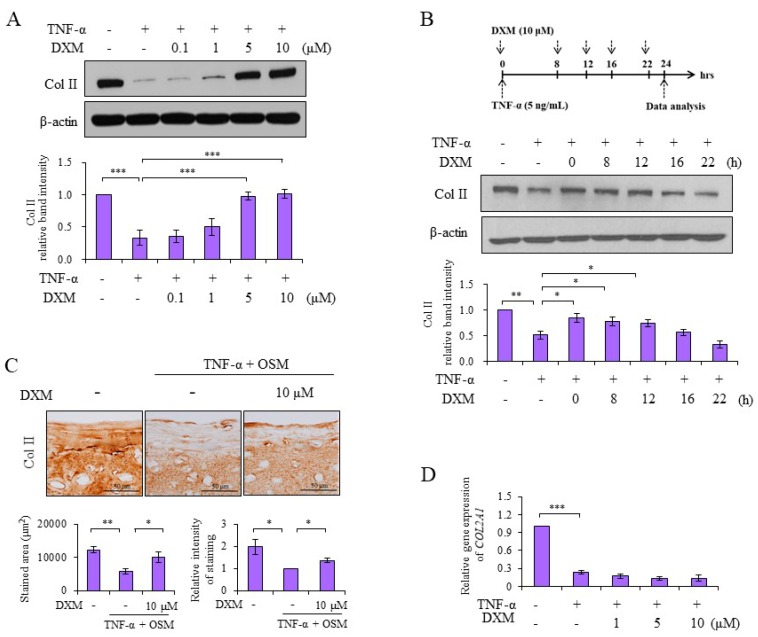
Dextromethorphan (DXM) reduced tumor necrosis factor-alpha (TNF-α)-induced loss of collagen II (Col II). (**A**) Porcine chondrocytes in serum-free medium were co-treated with TNF-α (5 ng/mL) and various doses of DXM as indicated for 24 h. Total cell lysates were analyzed with a Western blot. (**B**) Similar to (**A**), chondrocytes were treated with 10 μM DXM after TNF-α (5 ng/mL) stimulation at the indicated time, and total TNF-α stimulation time were 24 h (all TNF-α + samples). The total cell lysates were analyzed by Western blots. (**C**) Cartilage explants were stimulated with TNF-α (5 ng/mL) and Oncostatin M (OSM) (10 ng/mL), and treated with 10 μM DXM for 10 days. The retained Col II in cartilage explants were analyzed by immunostaining (original magnification, ×200), and the staining intensity and stained areas were calculated. (**D**) Chondrocytes were treated with TNF-α (5 ng/mL) and various doses of DXM as indicated for 16 h. Cellular mRNA were isolated and analyzed by real-time RT-PCR. Data were shown as the representative data from at least three independent experiments of different donors. Values are the means ± S.E.M. and significance (* *p* < 0.05; ** *p* < 0.01; *** *p* < 0.001) was analyzed by one-way ANOVA or Student’s *t*-test. The symbol of plus (+) or minus (−) indicates presence or absence of treatment, respectively.

Chondrocytes were treated with TNF-α and various doses of DXM simultaneously for 24 h, and the expression of colagen II (Col II) was measured by Western blotting. We observed that DXM dose-dependently preserved Col II levels in counteracting TNF-α-mediated Col II reduction (Figure 1A). When DXM was added simultaneously or at various time points after introducing the stimulant TNF-α, the chondroprotective effects of DXM on Col II expression could still be detected even 12 h after TNF-α stimulation (Figure 1B). We then prepared porcine cartilage explants of equal sizes to evaluate the chondroprotective effects of DXM ex vivo. As shown in Figure 1C, the loss of Col II was detectable in TNFα and Oncostatin M (OSM)-stimulated cartilage explants, as previously reported [12], and DXM prevented TNF-α+ OSM-mediated reduction of Col II. The semiquantitative PCR analysis showed that DXM had no effects on downregulated expression of Col II mRNA by TNF-α treatment (Figure 1D). These data suggested that the inhibitory effects of DXM on TNF-α-mediated Col II degradation might occur through blocking the mediators destroying Col II rather than through promoting the de novo synthesis of Col II.

### 2.2. DXM Decreased TNF-α-Induced MMP-13 Expression in Chondrocyte Cultures, 3-D Alginate Beads, and Cartilage Explants

**Figure 2:**
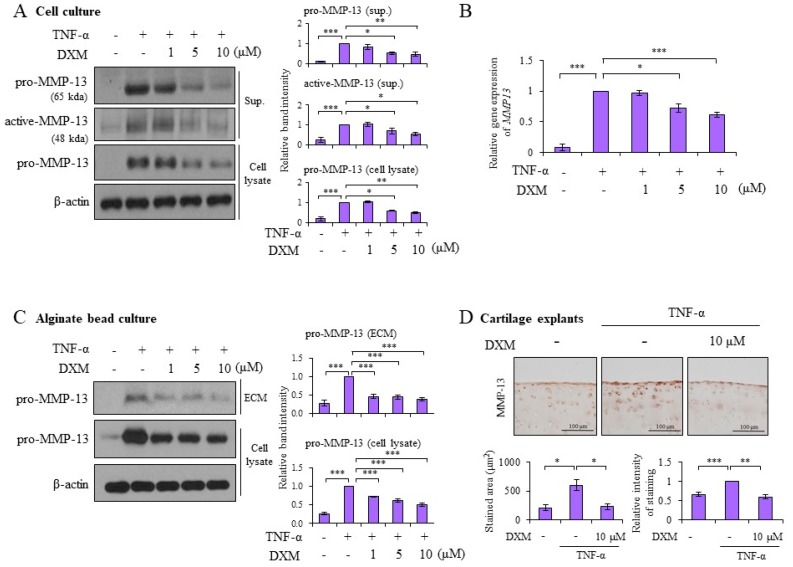
DXM repressed TNF-α-induced matrix metalloproteinases (MMP)-13. (**A**) Porcine chondrocytes were treated with TNF-α (5 ng/mL) and various doses of DXM as indicated for 24 h. Total cell lysate and supernatants (Sup.) were collected for analysis of pro-MMP-13 and active-MMP-13 expression by Western blotting. (**B**) Porcine chondrocytes were treated with TNF-α (5 ng/mL) and various doses of DXM for 8 h. Cellular mRNA were analyzed by real-time RT-PCR. (**C**) Porcine chondrocytes cultured in alginate beads were treated with TNF-α (5 ng/mL) in the presence of various doses of DXM for 72 h. MMP-13 levels were evaluated in cell lysate and extracellular matrix (ECM) with Western blots. (**D**) Cartilage explants were treated with TNF-α (5 ng/mL) in the presence or absence of DXM (10 μM) for 7 days. Expression of MMP-13 in cartilage explants was analyzed by immunostaining (original magnification ×100) and the staining intensity and stained areas were calculated. Data are shown as representative data from at least three independent experiments of different donors. Values are the means ± S.E.M. and significance (* *p* < 0.05; ** *p* < 0.01; *** *p* < 0.001) was analyzed by one-way ANOVA or Student’s *t*-test. The symbol of plus (+) or minus (−) indicates presence or absence of treatment, respectively.

Matrix metalloproteinases (MMP)-13 has been recognized as the major enzyme responsible for Col II degradation [13]. The effects of DXM on TNF-α-induced MMP-13 were examined. As shown in Figure 2A, DXM significantly decreased TNF-α-induced pro-MMP-13 levels in both cell lysates and supernatants. Meanwhile, the expression of the active form of MMP-13 was decreased by DXM treatment (Figure 2A). DXM also significantly inhibited TNF-α-induced MMP-13 (Figure 2B) as well as MMP-1 and MMP-3 (Figure S1) mRNA expression. Similar to the effects on Col II expression, DXM effectively inhibited pro-MMP-13 expression and MMPs RNA level even when it was administered 12 h after the addition of TNF-α stimulation (Figure S2). To avoid the effects of chondrocyte de-differentiation in a monolayer culture, we encapsulated chondrocytes in three-dimensional (3-D) alginate beads to preserve the chondrocytic phenotype [14]. DXM significantly decreased TNF-α-induced pro-MMP-13 expression in samples collected from ECM and cell lysates (Figure 2C). To further investigate the chondroprotective effects of DXM in cartilage, we prepared and examined porcine cartilage explants of equal sizes. Immunohistochemical staining confirmed that DXM inhibited TNF-α-induced pro-MMP-13 expression in cartilage explants (Figure 2D). Additionally, DXM also prevented IL-1-induced loss of Col ll and inhibited IL-mediated MMP-13 production (Figure S3). We further show that the anti-inflammatory effects of DXM could be detected in TNF-α-stimulated synoviocytes demonstrating the reduction of mRNA expression of pro-inflammatory cytokines or mediators, including IL-6, interferon-gamma-inducible protein 10 (IP-10), MMP-1, and MMP-3 (Figure S4).

### 2.3. Signaling Pathway Targeted by DXM

**Figure 3:**
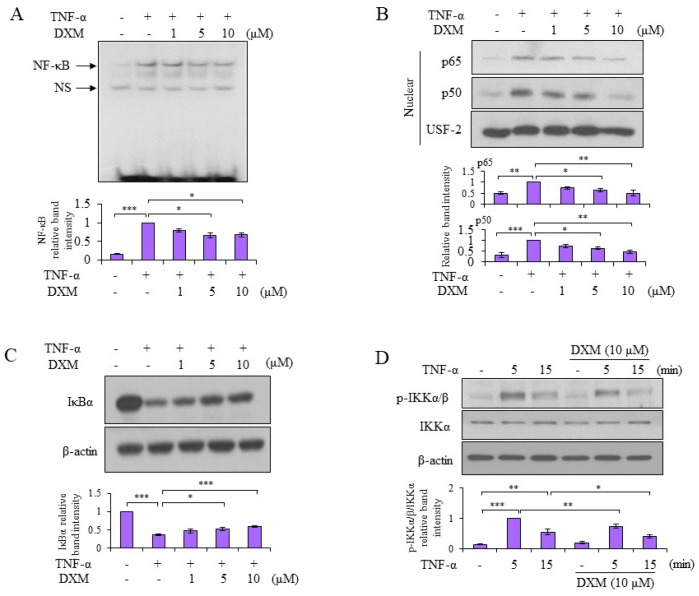
DXM suppressed TNF-α-induced nuclear factor-kappaB (NF-κB) signaling. (**A**) Porcine chondrocytes were treated with TNF-α (5 ng/mL) and various doses of DXM. Collected nuclear extracts were determined for DNA-binding activity of NF-κB by electrophoretic mobility shift assay (EMSA). (**B**,**C**) Porcine chondrocytes were pretreated with various doses of DXM for 2 h and then stimulated with TNF-α for 15 min. The nuclear extracts (**B**) and total cell lysates (**C**) were analyzed with Western blots. (**D**) Similar to (**C**), porcine chondrocytes were pretreated with 10 μM DXM for 2 h and then stimulated with TNF-α for 5 or 15 min. Total cell lysates were analyzed by Western blots. The results included a pool of at least three independent experiments from different donors and are shown as fold induction compared to the TNF-α-stimulated sample. Values are the means ± S.E.M. and significance (* *p* < 0.05; ** *p* < 0.01; *** *p* < 0.001) was analyzed by one-way ANOVA or Student’s *t*-test. NS: non-specific.

**Figure 4:**
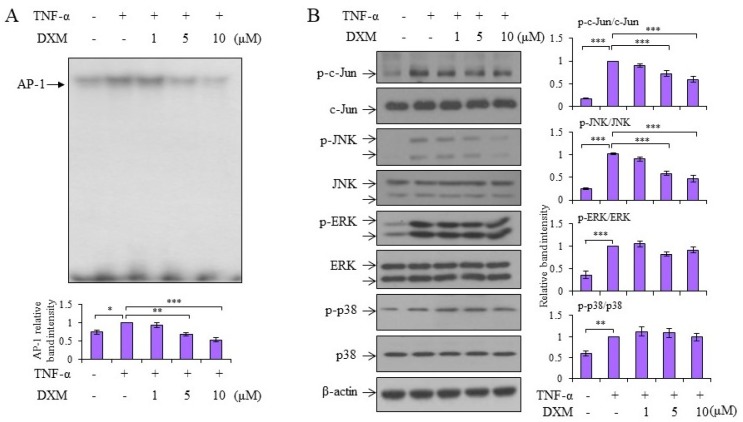
DXM inhibited TNF-α-induced activator protein-1 (AP-1) DNA binding activity through modulating c-Jun activation. (**A**) Porcine chondrocytes were treated with TNF-α (5 ng/mL) and various doses of DXM for 4 h and nuclear lysates were collected to determine the DNA-binding activity of AP-1 by EMSA. (**B**) Porcine chondrocytes were pretreated without or with various doses of DXM for 2 h, and then stimulated with TNF-α for 15 min. Total cell lysates were analyzed by Western blots. The results showed representative data from a pool of at least three independent experiments from different donors, and the data are shown as fold induction compared to the TNF-α-stimulated sample. Values are the means ± S.E.M. and significance (* *p* < 0.05; ** *p* < 0.01; *** *p* < 0.001) was analyzed by one-way ANOVA.

Both nuclear factor-kappaB (NF-κB) and activator protein-1 (AP-1)-signaling pathways, which are two major factors in inflammatory responses, are involved in TNF-α-induced MMP-13 expression [15]. The molecular mechanisms underlying the anti-inflammatory effects of DXM were examined. As shown in Figure 3A, DXM inhibited TNF-α-induced NF-κB DNA binding activity. In support of those results, the TNF-α-stimulated nuclear levels of both p65 and p50 decreased after DXM treatment (Figure 3B). Meanwhile, DXM counteracted TNF-α-mediated IκBα degradation and retained higher levels of IκBα in the cytosol (Figure 3C). The mechanism is likely to involve the downregulation of TNF-α-induced IκBα kinases, IKKα/β, by DXM (Figure 3D). In addition to the NF-κB signaling pathway, DXM inhibited TNF-α-induced AP-1 DNA binding activity (Figure 4A). The investigation into the upstream kinases mitogen-activated protein kinases (MAPKs) that regulate AP-1 activation revealed that DXM selectively inhibited TNF-α-induced expression of phosphorylated JNK and c-Jun (an indicator of c-Jun N-terminal kinase activity), and had no effect on expression of phosphorylated extracellular-signal-regulated kinase (ERK) or p38 (Figure 4B).

### 2.4. Effects and Mechanisms of DXM on TNF-α-Stimulated Human OA Chondrocytes

**Figure 5:**
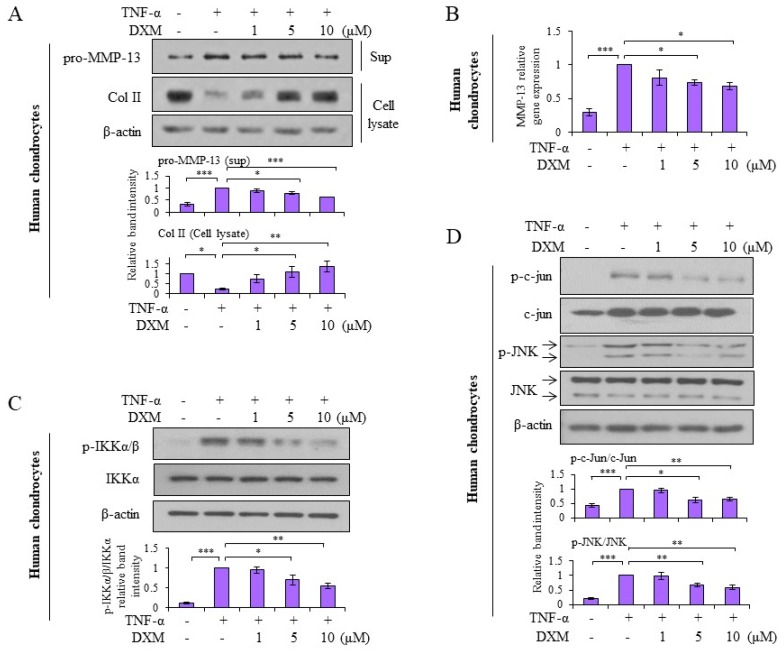
Mechanisms and chondro-protective effects of DXM were verified in human OA chondrocytes. (**A**) Human OA chondrocytes were co-treated with TNF-α (5 ng/mL) and various doses of DXM for 24 h. Total cell lysate and supernatants were analyzed for pro-MMP-13 and Col II expression with Western blots. (**B**) Chondrocytes were treated with TNF-α (5 ng/mL) and various doses of DXM for 8 h. The mRNA levels of MMP-13 were determined by real-time RT-PCR. (**C**) Chondrocytes were pretreated with various doses of DXM for 2 h, and then the cells were stimulated with TNF-α for 15 min. Total cell lysates were analyzed with Western blots (for pIKKα/β, IKKα and β-actin expression). (**D**) In parallel, the expression of p-c-Jun, c-Jun, p-JNK, and JNK (c-Jun N-terminal kinase) in total cell lysates was determined. Data were shown as representative data from at least three independent experiments of different donors. Values are the means ± S.E.M. and significance (* *p* < 0.05; ** *p* < 0.01; *** *p* < 0.001) was analyzed by one-way ANOVA.

Human chondrocytes were obtained and prepared from OA patients receiving total knee replacement, and the effects of DXM observed in porcine chondrocytes were verified in human chondrocytes. As shown in Figure 5A, DXM inhibited TNF-α-induced pro-MMP-13 expression and counteracted TNF-α-mediated Col II reduction. In addition, DXM suppressed TNF-α-induced MMP-13 mRNA expression (Figure 5B). Consistent with the results observed in porcine chondrocytes, DXM downregulated TNF-α-induced IKKα/β activation (Figure 5C) and phosphorylated c-Jun and JNK expression (Figure 5D).

### 2.5. DXM Successfully Inhibited Progression and Severity in CIA Mice

**Figure 6:**
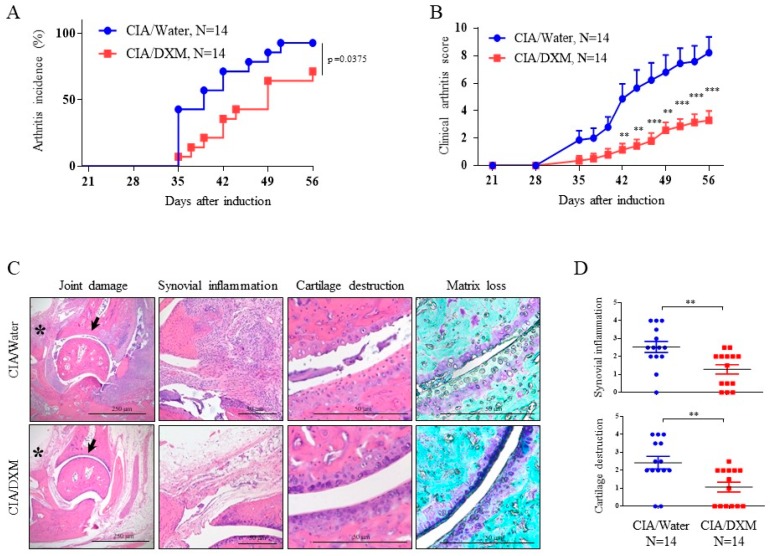
DXM delayed disease onset and severity in collagen-induced arthritis (CIA). Arthritis incidence (**A**) and total clinical arthritis score (**B**) were measured during the observation period of DXM (40 mg/kg/day)-treated CIA mice (*n* = 14) or water-treated CIA mice (*n* = 14). (**C**) Histological analysis of the joints of CIA mice treated with DXM or water were performed by hematoxylin and eosin (H&E) staining (original magnification, ×40 and ×200) and toluidine blue staining (original magnification, ×200) of the ankle joints. The asterisks point to the area of synovium and the arrows indicate the cartilage. (**D**) The histopathology scores on synovial inflammation and cartilage destruction were quantitatively analyzed. Values are the means ± S.E.M. and significance (** *p* < 0.01; *** *p* < 0.001) was analyzed by Kaplan–Meier log-rank analysis (**A**), two-way ANOVA (**B**), and the Mann-Whitney U test (**D**).

**Figure 7:**
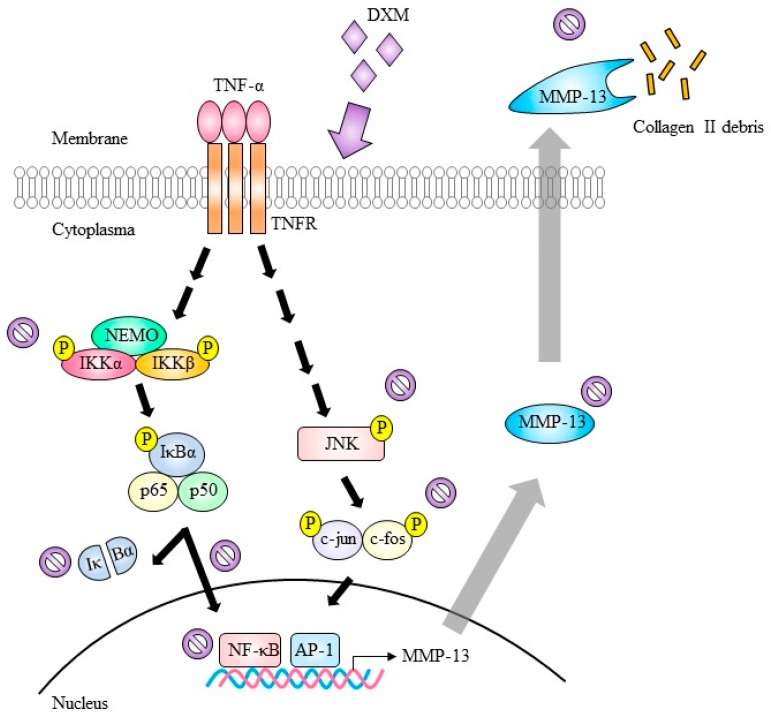
Depicted mechanisms showing how DXM mediates anti-inflammation through blocking TNF-α-induced MMP-13 expression and Col II degradation. Under TNF-α stimulation, the NF-κB and AP-1 signaling are activated to promote MMP-13 transcription. Enhanced expression of MMP-13 was secreted to cause destruction of extracellular matrix elements, such as collagen II. With DXM treatment, both IKK-IκBα-NF-κB and JNK-AP-1 signaling pathways were blocked, and MMP-13 expression was decreased to attenuate TNF-α-induced damage of collagen II. The DXM treatment can thus provide a beneficial effect for treatment of osteoarthritis and possibly other autoimmune arthritis. NEMO: NF-κB essential modulator. 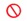 indicate inhibition.

We evaluated the effects of DXM in a collagen-induced arthritis (CIA) mouse model to extend the potential applications of this knowledge. The induction of CIA was accomplished through intradermal injection of bovine Col II at the base of tail in mice, and DXM at the dosage of 40 mg/kg/day, according to the report by other researchers [9], was administered daily through nasogastric tube feeding shortly after we immunized DBA1J mice. Total experimental period were 56 days with careful examination on disease progression every two to three days. As shown in Figure 6A, DXM treatment significantly reduced the incidence of CIA. Based upon the clinical scoring system [16], DXM significantly attenuated the disease severity (Figure 6B). With histological analysis by hematoxylin and eosin (H&E) staining as well as toluidine blue staining, the results showed that DXM significantly alleviated both cartilage destruction and synovial inflammation (Figure 6C,D). In addition, DXM protected collagen II from degradation and decreased MMP-13 expression are shown with immunochemistry (Figure S5). We found no significant difference in body weight in both the therapeutic groups and the controls (Table S1). Collectively, the in vitro, ex vivo, and in vivo results suggest the potential benefit of DXM in the treatment of joint inflammation through chondroprotective and anti-inflammatory effects. A simplified cartoon illustrates the mechanisms regarding how DXM works in chondrocytes (Figure 7).

## 3. Discussion

Therapeutic agents targeting TNF-α have been developed and used to treat patients with autoimmune arthritis, such as psoriatic arthritis, ankylosing spondylitis, and rheumatoid arthritis (RA) [17]. TNF-α mediates many different aspects of inflammation that cause joint damage, and the activation of MMP-13 expression and suppression of Col II are only some of these mechanisms. It is likely that the anti-inflammatory effects of DXM can also be demonstrated in TNF-mediated inflammatory bone remodeling and rheumatoid arthritis [18,19]. As we aimed to explore the possible protection effect of DXM “on cartilage”, to meet this demand, fresh porcine joints are a source easily available and are able to isolate enough amounts of primary chondrocytes, which are adequate for cellular and molecular analysis, and the comparable results on human chondrocytes were represented the possible clinical application. Both porcine and human chondrocyte shows that while TNF-α-mediated reduction of Col II can occur both by suppressing Col II protein levels and inhibiting Col II mRNA expression, the effects of DXM were only observed to counteract the suppression of protein and had no effect on Col II mRNA expression. This finding was comparable to the suppression of MMP-13 by DXM. Interestingly, in contrast to the effects on Col II, both protein and mRNA levels of MMP-13 were downregulated by DXM. These results suggest that MMP-13 might be a direct or indirect target for DXM-mediated chondroprotective effects.

Because the activation of both NF-κB and AP-1 signaling pathways plays a crucial role in mediating OA cartilage destruction, the suppression of these two signaling pathways by DXM suggests this drug is a very powerful protective agent against OA pathogenesis [20,21]. It is also interesting to find that DXM preserved certain types of specificity by targeting JNK activity but not other MAPKs, such as ERK and p38. Although our studies demonstrated that DXM could inhibit TNF-α-induced IKKα/β-IκBα-NF-κB and JNK-AP-1 activation, these experiments could not confirm the exact targets of DXM. Besides, DXM was effective on prevent TNF mediated Col II reduction and TNF induced MMP-13 expression even 12 h after TNF-α stimulation. These results demonstrated the possible therapeutic benefits of DXM after inflammation already been initiated. In Figure S2, DXM still efficiently inhibited MMPs RNA level after TNF stimuli 12 h, although the protective effects were not that effective compared with co-treatment of DXM for 24 h. We propose that in addition to NF-κB and AP-1 signaling, alternative cellular signaling such as miRNA might also be involved. Further studies are needed to help identify the real targets of DXM.

Several different animal models have been generated for studying OA pathogenesis; however, the commonly used traumatic OA model in which an anterior cruciate ligament transection is performed is representative of only a very limited number of OA populations. Because the cartilage damage shares certain similarities between OA and RA [2], the commonly used RA animal model named CIA was chosen to examine the in vivo effects of DXM, as we reported earlier [14]. In this CIA model, many pro-inflammatory cytokines, such as IL-1β, TNF-α, and IL-6, are also increased in both serum and synovial fluid in patients with RA and likely severe OA that play crucial roles in mediating the pathogenesis of these diseases [22]. The chondroprotective and anti-inflammatory effects of DXM were fully supported in this animal study by observing that DXM treatment reduced the incidence of CIA as well as decreased disease severity. Using H&E staining, we observed that treatment with DXM protected against cartilage damage and bone erosion. In addition to cartilage damage, synovial inflammation is also a hallmark of OA [23]. The anti-inflammatory and chondroprotective effects of DXM were further extended through suppressing TNF-α-induced synovial inflammation by inhibiting the levels of pro-inflammatory cytokines, such as IL-6 and IP-10, as well as suppression of cartilage destruction-inducing enzymes MMP-1 and MMP-3. Most recently, inflammations play a critical role and recognized as a driver on OA pathogenesis process on varied type of OA [24,25,26]. In addition to TNF-α stimulation, IL-1β, the common stimulant in OA disease model, were also used in this study to verify the benefit effects of DXM. Similar protective response has been shown with IL-1β stimulation that DXM blocked IL-1β-stimulated Col II degradation and IL-1β-induced MMP-13 expression as TNF-α stimulation. These data suggested that DXM might be able to challenge different inflammatory stimuli in OA and RA disease progression. Altogether, the results of our in vivo studies examining CIA mice correlate with the results observed in in vitro studies of porcine chondrocytes and human primary chondrocytes.

Several NMDA receptor subunits, including 2A, 2B, and PSD-95, have been detected in both normal and human OA chondrocytes, except that normal chondrocytes do not express the NR2B subunit [27]. Accordingly, there are also subtle difference in the NMDA-mediated signaling between normal and OA [27]. Early studies also showed that NMDA receptor-mediated signaling may modulate the cellular response to mechanical stimulation as well as mechanotransduction [28]. The knock-down of the NR1 subunit of NMDA receptors or pharmacological blockade with [^3^H]MK-801, which is a specific NMDA receptor antagonist, were shown to inhibit chondrocyte proliferation [29]. Magnesium deficiency has been implicated as one of the potential mechanisms leading to OA pathogenesis. Under conditions of magnesium deficiency, several effects, such as increased production of inflammatory mediators, cartilage damage, defective chondrocyte biosynthesis, and aberrant calcification have been observed [30]. The intra-articular administration of MgSO_4_ resulted in attenuation of cartilage degeneration and synovitis as well as led to the improvement of mechanical allodynia and thermal hyperalgesia in an experimental rat OA model [31]. The mechanisms have been shown to be mediated through inhibition of phosphorylation of cellular NMDA receptor phosphorylation and apoptosis [31]. By examining the molecular mechanisms of anti-inflammatory and chondroprotective effects of anti-NMDA compound DXM, this study further expanded our knowledge regarding the roles of NMDA in inflammatory arthritis.

Choices of very effective therapeutics against OA remain to be challenging and new concept and new approaches are emerging [11,32]. For this, early prevention of cartilage loss and stimulating regrowth of lost cartilage is becoming more and more important [33,34]. It has been suggested that components from food or drink or even dietary supplements that preserve modest immunomodulatory properties while having limited or no adverse events, such as resveratrol, may be reasonable alternatives for long-term anti-OA therapies [35]. In addition to these choices, repurposing medications with long-term safety profiles to reduce or alleviate inflammation may also be a good strategy for treating OA. To provide solid evidence supporting the potential of DXM as an anti-OA regimen, we choice the most straightforward and powerful parameters to study the mechanisms, namely, TNF-α as the stimuli and NF-κB, AP-1, Col II, and MMP-13 as the readouts, as well as the CIA animal model. The present study demonstrated novel benefits of DXM that could potentially make DXM useful as an anti-OA drug. Further clinical trials with DXM may be considered as an alternative therapeutic agent for the management of patients with OA or even RA, given its reliable safety profile.

## 4. Materials and Methods

### 4.1. Reagents and Antibodies

The recombinant human TNF-α, IL-1β, and OSM were purchased from R&D (Canandaigua, NY, USA). The purified compound DXM was obtained from Sigma-Aldrich Chemical Company (St. Louis, MO, USA). Antibodies against MMP-13, IκBα, p65, p50, USF-2, IKKα, c-Jun, ERK, p38, and JNK were purchased from Santa Cruz Biotechnology (Santa Cruz, CA, USA). Antibodies against c-Jun, p-IKKα/β, p-c-Jun, p-ERK, p-p38, and p-JNK were purchased from Cell Signaling (Danvers, MA, USA). Antibodies against Col II were purchased from Chemicon International (Temecula, CA, USA). Unless otherwise specified, all other reagents were purchased from Sigma-Aldrich Chemical Company.

### 4.2. Isolation and Culture of Human and Porcine Chondrocytes

After obtaining written informed consent and prior approval from the Institutional Review Board of the Tri-Service General Hospital (Protocol No. TSGH-C103-070, 2 September 2013), cartilage from OA patients receiving total knee joint replacement was obtained aseptically. The preparation of chondrocytes from human cartilage was performed as previously described [36]. In brief, full-thickness articular cartilage was removed from the underlying bone and cut into pieces. After enzymatic digestion with 2 mg/mL protease (Sigma) in Dulbecco’s modified Eagle’s medium (DMEM) containing 10% fetal bovine serum (FBS)/antibiotics (Invitrogen, Carlsbad, CA, USA) for 1 h, the specimens were digested with 2 mg/mL collagenase I and 500 U/mL hyaluronidase in DMEM medium containing 10% FBS/antibiotics overnight. After passaging the cells through a cell strainer, the cells were collected and seeded in T75 flasks in medium containing 10% FBS for 2 to 3 days before use. Porcine cartilage was obtained from the hind leg joints of pigs. The method for preparation of chondrocytes from porcine cartilage was similar to the preparation of chondrocytes from human cartilage [14]. When cultured in a monolayer, chondrocytes de-differentiate into fibroblast-like cells after a few generations of passage [37,38]. To avoid this issue, the chondrocytes used throughout this study were maintained for one passage so that the cells retained the shape and characteristics of chondrocytes [14].

### 4.3. Preparation of Porcine Synoviocytes

Porcine synoviocytes were prepared similarly to the preparation for human fibroblast-like synoviocytes according to the report from other researchers [39]. In brief, the synovial tissue in pig joints was obtained aseptically and digested with 0.25% trypsin-EDTA (Gibco Life Technologies, Gaithersburg, MD, USA) for 30 min and then incubated with 0.4 mg/mL collagenase in DMEM containing 10% FBS/antibiotics for 3 h. After enzymatic digestion, the cells were passaged through a cell strainer and cultured in DMEM containing 10% FBS/antibiotics. After additional 3 or 4 passive passages, the p3 to p4 cells were used for experiments.

### 4.4. Preparation of Cartilage Explants

The experiments with cartilage explants has been described in our previous report [14]. Briefly, articular cartilage specimens of equal sizes were prepared from the joint located close to the femoral head of the pig hind limb with a stainless-steel dermal punch (diameter, 3 mm; Aesculap, Tuttlingen, Germany). The samples were weighed thereafter. Each cartilage explant was then placed in a 96-well plate in the culture medium with DMEM containing antibiotics and 10% FBS for 24 h. After culture with serum-free DMEM, the cartilage explants were used for experiments.

### 4.5. Analysis by a Real-Time Polymerase Chain Reaction with Reverse Transcription

Total RNA was prepared using TRIzol reagent (Invitrogen; Carlsbad, CA, USA) according to our previous report [14]. Reverse transcription was carried out in a 20 μL mixture containing 2 μg of total RNA, random hexamers, 10× RT buffer, a mixture of dNTP (Promega; Madison, WI, USA), and Moloney Murine Leukemia Virus Reverse Transcriptase following the manufacturer’s protocol (Invitrogen). After reverse transcription of RNA to cDNA, the cDNA samples were subjected to PCR reactions (power SYBR Green PCR Master Mix, Applied BioSystems, Foster City, CA, USA). In brief, 10 ng of cDNA were amplified in a 20 μL mixture containing 1× Master Mix and 100 nM gene-specific primers. The sequences of individual primers are shown in Table S2 [40,41]. There were more than 40 cycles of PCR reaction with 95 °C for denaturation and 60 °C for annealing and extension on a Roche LightCycler 480 (Roche, Basel, Switzerland).The changes in gene expression caused by stimulation with TNF-α in the presence or absence of DXM were calculated with the following formula: fold change = 2^−∆ (∆*C*t)^, where ∆*C*_t_ = *C*_t targeted gene_ − *C*_t GAPDH_, and ∆ (∆*C*_t_) = ∆*C*_t stimulated_ − ∆*C*_t control_.

### 4.6. Western Blotting

Enhanced chemiluminescence Western blotting (Amersham-Pharmacia, Arlington Heights, IL, USA) was performed as described previously [14]. Briefly, equal amounts of protein prepared from lysing cells with RIPA buffer or equal volume of supernatants with or without concentration using Amicon Ultra spin columns (EMD Millipore, Billerica, MA, USA) were analyzed using sodium dodecyl sulfate–polyacrylamide gel electrophoresis (SDS-PAGE) and transferred to a nitrocellulose filter. To do immunoblotting, the nitrocellulose filter was incubated with Tris-buffered saline containing 1% Triton X-100 and 5% non-fat milk for 1 h and then the filter was blotted with protein-specific antibodies for another 2 h at room temperature.

### 4.7. Nuclear Extract Preparation

Nuclear extracts from chondrocytes were prepared as described previously [42]. Briefly, the cells (3–4 × 10^6^) were mixed with 50 μL buffer A (10 mM HEPES, pH 7.9, 10 mM KCl, 1.5 mM MgCl_2_, 1 mM dithiothreitol (DTT), 1 mM PMSF, and 3.3 μg/mL aprotinin) and left for 15 min at 4 °C with occasional gentle vortexing. After centrifugation at 14,000 rpm (15,000 *g*) for 3 min, the supernatants were removed. The pelleted nuclei were washed with 50 μL of buffer A once and then resuspended in 20 μL of buffer C (20 mM HEPES, pH 7.9, 420 mM NaCl, 1.5 mM MgCl_2_, 0.2 mM EDTA, 25% glycerol, 1 mM DTT, 0.5 mM PMSF, and 3.3 μg/mL aprotinin). The samples were then incubated for 30 min at 4 °C with occasional vigorous vortexing. After centrifugation at 14,000 rpm for 30 min, the supernatants were collected and used as nuclear extracts.

### 4.8. Electrophoretic Mobility Shift Assay (EMSA)

The EMSA was performed as described in our previous report [42]. Oligonucleotides containing a nuclear factor kappaB (NF-κB)- or activator protein-1 (AP-1)-binding site were purchased from Promega (Madison, WI, USA) and used as DNA probes. The T4 kinase (Promega) was used to radio-label the probes with [γ-32p]ATP. A mixture containing the radio-labeled probe, 5 μg nuclear extract and the binding buffer (10 mM Tris-HCl (pH 7.5), 50 mM NaCl, 0.5 mM EDTA, 1 mM DTT, 1 mM MgCl_2_, 4% glycerol, and 2 μg poly(dI-dC)) was prepared to proceed with the binding reaction for 20 min at room temperature. The final reaction mixture was analyzed in a 6% non-denaturing polyacrylamide gel, and 0.5× Tris/Borate/EDTA was used as an electrophoresis buffer.

### 4.9. 3-D Alginate Bead Experiments

The 3-D alginate bead experiments were performed according to our previous report [14]. In brief, chondrocytes were gently resuspended in alginate solution (1.2% low-viscosity alginate in 0.15 M NaCl) with a density of 3.75 × 10^6^ cells/mL. An automatic Pipetman was used to slowly drip the chondrocyte suspension (drop volume, 10 μL) into a CaCl_2_ solution (102 mM). After the mixing steps, the beads were allowed to completely polymerize for 10 min at room temperature. Then, the CaCl_2_ solution was removed and the beads were washed with normal saline. After this, the beads were cultured in DMEM containing 10% FBS at 37 °C with the supplement of 5% CO_2_. After treatment, the culture medium for the beads was replaced with iced normal saline. The beads were then transferred into Eppendorf tubes containing a cold 55 mM sodium citrate solution. The alginate beads were rotated for 30 min at 4 °C to dissolve the gel and to release the cells from the beads. The cells were then centrifuged at 12,000× *g* for 10 min and the supernatants were collected and used as the ECM fraction. After wash with cold normal saline, the cell pellet was lysed in a RIPA buffer. The ECM fractions and cell lysates were analyzed with the relevant assays.

### 4.10. Immunostaining of Porcine Cartilage

Cartilage explants were prepared and handles as described in our previous report [14]. The frozen cartilage explants were prepared as noncontiguous microscopic sections (7 μm) and then cut at −20 °C on a Microm cryostat (Thermo Fisher Scientific, Walldorf, Germany) and mounted on Superfrost Plus glass slides (Menzel-Gläser, Braunschweig, Germany). Immunohistochemistry staining was conducted to assess the expression of MMP-13 (Santa Cruz, CA, USA), and Col II (Chemicon International, Temecula, CA, USA) was performed according to our previous report with some modification [14]. The levels of targeted protein expression in images were analyzed using IMAGEJ software (National Institutes of Health, Bethesda, MD, USA).

### 4.11. Collagen-Induced Arthritis in Mice

The animal experiments were approved by the National Defense Medical Center Laboratory Animal Center (IACUC-15-080, 19 March 2015), Taiwan, and all mice were maintained and treated according to the institute’s guidelines. In addition, the experimental procedures in animal studies were performed following the guidelines and regulations of the institution. The murine CIA model was produced as described previously with minor modification [14]. The male DBA/1J mice (age, 8–10 weeks) were injected intradermally through tails with 100 μL of bovine Col II (1 mg/mL) and complete Freund’s adjuvant containing 2 mg/mL of *Mycobacterium tuberculosis*. For testing the effect of DXM, water or DXM at the dosage of 40 mg/kg/day, according to the report by other researchers [9], was administered daily through nasogastric tube feeding shortly after we immunized DBA1J mice. Thirty-five days after Col II injection, signs of inflammation in foot paws of the animals were closely monitored every 2–3 days. The 4-point scale was used to evaluate clinical disease activity in each paw: 0 points stands for no evidence of erythema and swelling; 1 point stands for erythema and mild swelling confined to the tarsals or ankle joints; 2 points stands for erythema and mild swelling extending from the ankle to the tarsals; 3 points stands for erythema and moderate swelling extending from the ankle to the metatarsal joints; and 4 points stands for erythema and severe swelling encompassing the ankle, foot and digits, or limb ankyloses [16]. The mice were sacrificed by inhaling CO_2_, and the foot paws were collected in 10% formalin to be fixed for pathological analysis.

### 4.12. Histological Analysis in CIA

Collected foot paws were decalcified in 20% EDTA and then embedded in paraffin. After sectioning in 3–5 μm size, the samples were stained with hematoxylin–eosin and toluidine blue. Scores for the pathological changes in diseased joints were based on the hematoxylin-eosin staining as described in previous studies [43]. The score is classified as follows. For inflammation, score 0 stands for no inflammation; score 1 stands for slight thickening of lining layer or some infiltrating cells in the sublining layer; score 2 stands for slight thickening of lining layer plus some infiltrating cells in the sublining layer; score 3 stands for thickening of the lining layer, influx of cells in the sublining layer, and presence of cells in the synovial space; and score 4 stands for synovium highly infiltrated with many inflammatory cells. For cartilage damage, score 0 stands for no destruction; score 1 stands for minimal erosion limited to single spots; score 2 stands for slight to moderate erosion in a limited area; score 3 stands for more extensive erosions; and score 4 stands for general destruction. To do immunohistochemistry for MMP-13 and Col ll, according to previous study [44], we deparaffinized and rehydrated the paraffin-embedded sample. After citrate antigen retrieving, we performed immunochemistry using the Ultravision Quanto De-tection System HRP Polymer kit (Thermo Fisher Scientific, Waltham, MA, USA).

### 4.13. Statistical Analysis

Statistical analysis was performed using GraphPad Prism v.6.01 software (GraphPad Software Inc., San Diego, CA, USA). The statistical significance between two groups was determined by Student’s *t*-test, and when multiple groups were analyzed, one-way ANOVA with the Bonferroni’s post-hoc comparisons were chosen to examine whether the interaction effects were significant. The incidence curves of CIA mice were evaluated using the Kaplan-Meier log-rank analysis, and the clinical scores of CIA mice were recorded and analyzed using two-way ANOVA with Bonferroni’s post-hoc multiple comparison tests. Histological analysis was performed and measured with a Mann–Whitney U test. Data are displayed as means ± S.E.M. and the *p* values less than 0.05 were considered to be statistically significant (* *p* < 0.05; ** *p* < 0.01; *** *p* < 0.001).

## Supplementary Materials

Supplementary materials can be found at http://www.mdpi.com/1422-0067/19/3/825/s1.

## Abbreviations

AP-1	activator protein-1
CIA	collagen-induced arthritis
Col II	collagen II
DXM	dextromethorphan
ECM	extracellular matrix
ERK	extracellular-signal-regulated kinase
IKK	I kappa B kinase
IP-10	interferon-gamma-inducible protein 10
JNK	c-Jun N-terminal kinase
MAPK	mitogen-activated protein kinase
MMP	matrix metalloproteinases
NF-κB	nuclear factor-kappaB
NMDA	*N*-methyl-d-aspartate
OA	osteoarthritis
OSM	oncostatin M
RA	rheumatoid arthritis
TNF-α	tumor necrosis factor-alpha
