# Supplementary material for: Chondroprotective Effects and Mechanisms of Dextromethorphan: Repurposing Antitussive Medication for Osteoarthritis Treatment
Source: Int J Mol Sci. 2018 Mar 12;19(3):825. doi: 10.3390/ijms19030825 (PMC5877686; doi:10.3390/ijms19030825)
Supplement: Supplementary file 1 [file ijms-19-00825-s001.pdf]

Supplementary information

Title: Chondroprotective effects and mechanisms of dextromethorphan: Repurposing antitussive medication for osteoarthritis treatment

Authors: Liv Weichien Chen, Feng-Cheng Liu, Li-Feng Hung, Chuan-Yueh Huang, Shiu-Bii Lien, Leou-Chyr Lin, Jenn-Haung Lai and Ling-Jun Ho

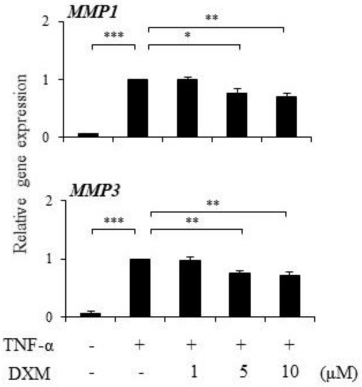

Figure S1. The inhibitory effects of DXM on TNF-α-induced MMP-1 and -3 mRNA in porcine chondrocytes. Porcine chondrocytes were treated with TNF-α (5 ng/ml) without or with various doses of DXM for 8 h. Cellular mRNA was prepared and analyzed by real time RT/PCR. Data are shown as representative data from at least three independent experiments with different donors. Values are the means ± S.E.M. and significance was analyzed by one-way ANOVA. (*p < 0.05; **p < 0.01; ***p < 0.001)

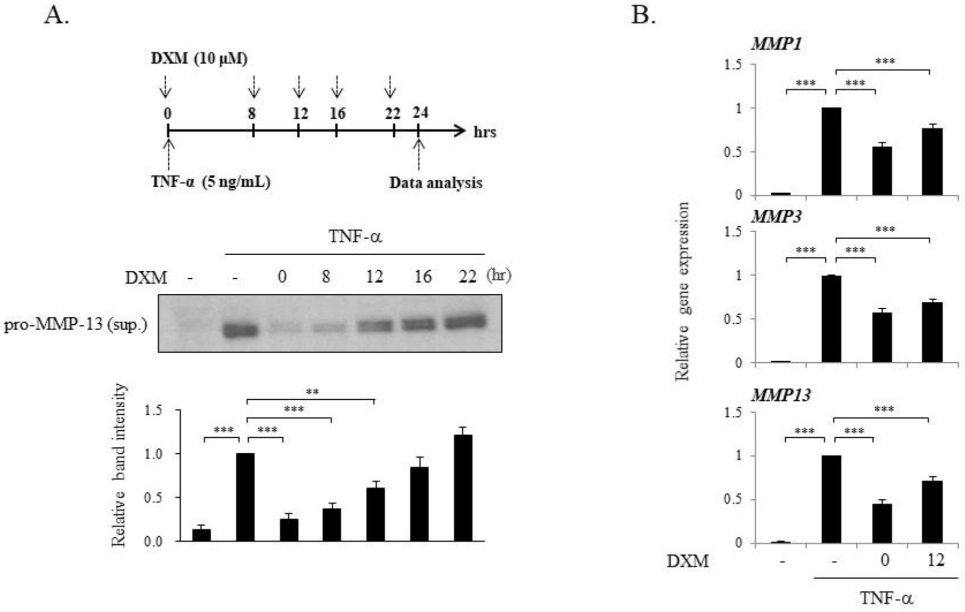

Figure S2. The effects of post-stimulation treatment of DXM on TNF-α induced MMP-13 expression in porcine chondrocytes. (A) Porcine chondrocytes in serum-free medium were treated with 10 μM DXM after TNF-α (5 ng/ml) stimulation at the indicated time points, and the collected supernatants were analyzed for MMP-13 expression with Western blots. (B) Similar to (A), after indicated treatment, cellular mRNA was prepared and analyzed by real time RT/PCR. Data are shown as representative data from at least three independent experiments from different donors. Values are the means ± S.E.M. and significance was analyzed with Student's t-test. (*p < 0.05; **p < 0.01; ***p < 0.001)

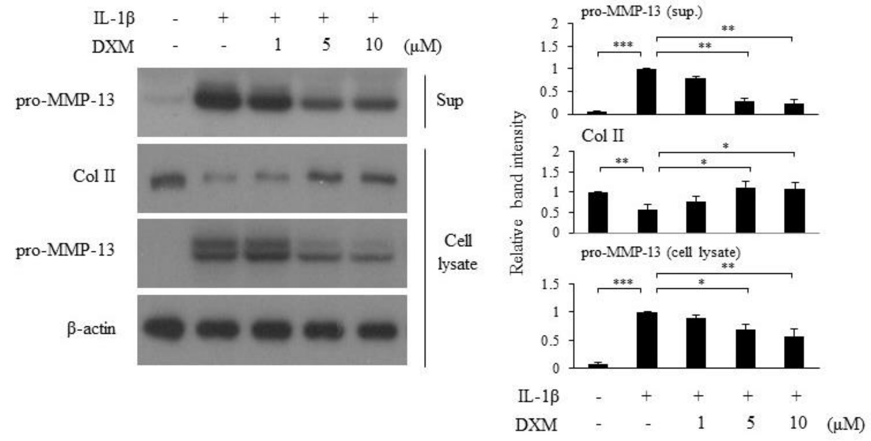

Figure S3. The benefit effects of DXM on IL-1β induction in porcine chondrocytes. Porcine chondrocytes were treated with IL-1β (40 ng/ml), with or without various doses of DXM for 24 h, and the total cell lysate and collected supernatants were analyzed with Western blots. Data are shown as representative data from at least three independent experiments from different donors. Values are the means ± S.E.M. and significance was analyzed with one-way ANOVA.. (*p < 0.05; **p < 0.01; ***p < 0.001)

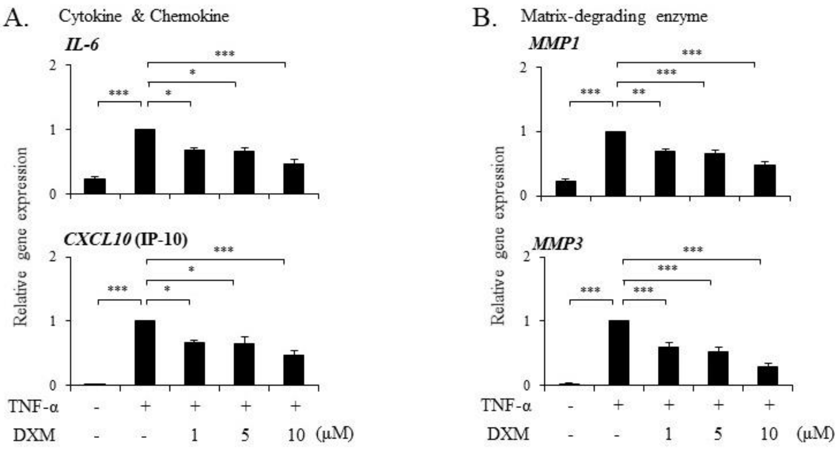

Figure S4. The inhibitory effects of DXM on TNF-α-induced cytokines and MMPs in porcine synoviocytes. (A and B) Porcine synoviocytes were prepared through passive passaging and were used for experiments in p3 to p4. Porcine synoviocytes were treated without or with various doses of DXM and then stimulated with TNF-α (5 ng/ml) for 4 h. The mRNA levels of interleukin-6 (IL-6), interferon-gamma-inducible protein 10 (IP-10, also named as CXCL10), MMP-1, and MMP-3 were measured. Data are shown as representative data from at least three independent experiments of different donors. Values are the means ± S.E.M. and significance was analyzed by one-way ANOVA. (*p < 0.05; **p < 0.01; ***p < 0.001)

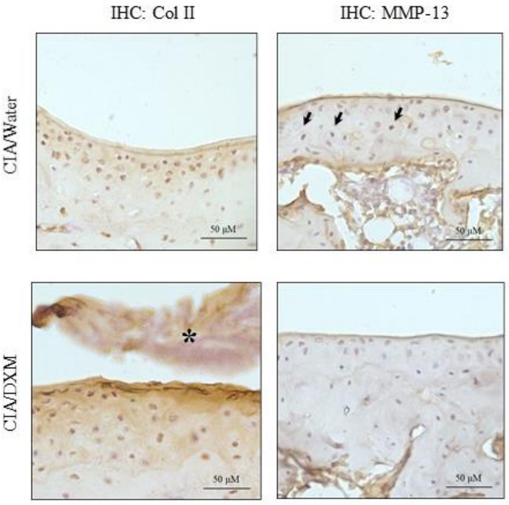

Figure S5. Represented data of immunochemistry Col II and MMP-13 of fig 6 (original magnification, ×200). The arrows indicate the staining of MMP-13 and asterisks indicate ignored mess.

CIA/Water									
DBA1J No.	0 days	7 days	14 days	21 days	28 days	35 days	42 days	49 days	56 days
31	22.2	22.6	23.6	23.3	24.3	26.5	26.2	26.6	25.4
33	24.8	25.4	25.8	26.4	27	28	27.6	22.9	25.3
34	22.6	22.8	23.2	23.9	24.2	25.5	24.2	23.7	24.1
35	23	23.6	24.2	24.3	24.6	24.4	23.1	25.1	24.4
36	26.2	26.6	27.4	28.7	29.4	23.4	22	23.7	25.7
55	21.4	21.6	21.7	22	22.4	22.9	22.3	21.1	22.8
59	21.2	21.4	21.8	21.8	22.4	22.9	22.8	20.6	22.6
62	21.8	21.4	21.6	22.2	23.4	25.1	22.4	18.2	20.8
72	20.3	19.7	20.1	20	21.2	22	22	20.8	20.5
73	18.6	18.8	19.5	19.9	20.3	20.7	21.2	21.2	21
74	20.7	19.7	20.1	22	21.2	20.3	20.2	21.6	21.2
77	22.5	23.1	22.5	23.1	24.2	24.6	24.6	24.1	23.9
80	23.9	23.4	23.1	24.3	23.6	24	24.6	23	23.3
81	22.8	21.8	22.1	23.7	23.8	24.7	24.4	23.5	23
AVG	22.29	22.28	22.62	23.26	23.71	23.93	23.40	22.58	23.14
SE	0.51	0.57	0.58	0.62	0.63	0.56	0.53	0.57	0.47

CIA/DXM									
DBA1J No.	0 days	7 days	14 days	21 days	28 days	35 days	42 days	49 days	56 days
38	22.6	22.2	23.4	24	24	20.5	21.2	22.8	24
39	21.5	22.3	22.8	23	23.4	23.1	22.9	24	22.2
41	22	22.4	22.6	22.9	23.4	24.1	23.2	22.2	22.8
42	23.5	24.6	25.2	26.3	26.6	27.7	27.1	26.2	26.4
56	20.2	19.8	20.6	21.6	22	22.5	22.4	21.7	22
63	20	20.4	20.4	20.8	22.2	24.3	23.9	22.8	23.6
66	20.4	20.6	20.8	20.6	21.3	22.4	20.5	19.2	20.7
70	17.8	18.2	18.8	19	19.6	20.2	20.6	21.3	20.6
71	21.2	20.6	22.1	23.1	23.6	24.1	24.4	25.5	24.8
75	21.9	20.5	20.8	21.6	22.4	23.9	24.6	24.6	23.9
76	22.9	22.8	23.1	24.3	23.8	24.3	25	26	25.2
78	21.8	22.5	23.4	24	24.7	25.2	25.3	24.2	24.3
79	23.1	22.8	22.9	24.9	25.3	25.8	25.6	26	23.7
82	21.3	20.5	19.7	21.3	22	23.4	23	24	24.3
AVG	21.44	21.44	21.90	22.67	23.16	23.68	23.55	23.61	23.46
SE	0.40	0.44	0.47	0.52	0.47	0.52	0.52	0.55	0.44

TTEST	0.205	0.257	0.345	0.478	0.492	0.748	0.842	0.207	0.623

Table S1. Weight changes in water- or DXM-treated CIA mice.

**Gene**	**GenBank Accession number**	Forward Primer (5'-3')	Reverse Primer (5'-3')
Porcine			
COL2A1	XM_001925959	GAGAGGTCTTCCTGGCAAAG	AAGTCCCTGGAAGCCAGAT
MMP1	NM_001166229	AAGCAGACATAATGATATCCTTTGTCA	TGAGCATCCCCTCCAATACCT
MMP3	NM_001166308	ACACCCTGGGTTTTCCTTCAACAGTA	CCTGGCTCCATGGATTGTCTCTTCTC
MMP13	XM_003129808	GATCCCCATTTTGATGATGATGAA	GTCTTCATCTCCTGGACCATAGAGAGA
IL-6	NM_001252429	TTCACCTCTCCGGACAAAAC	TCTGCCAGTACCTCCTTGCT
CXCL10	NM_001008691	CCCACATGTTGAGATCATTGC	CATCCTTATCAGTAGTGCCG
GAPDH	NM_001206359	TGGTGAAGGTCGGAGTGAAC	GCCACAGTTTCCCAGAGG
Human			
MMP13	NM_002427	TCCCAGGAATTGGTGATAAAGTAGA	CTGGCATGACGCGAACAATA
GAPDH	NM_001289745	AGGTGAAGGTCGGAGTCAAC	CCATGTAGTTGAGGTCAATGAAGG

Table S2. The primer sequences used in the real-time PCR experiments.
